# Biofilm Formation on the Surfaces of CAD/CAM Dental Polymers

**DOI:** 10.3390/polym15092140

**Published:** 2023-04-29

**Authors:** Stefan Vulović, Nataša Nikolić-Jakoba, Milena Radunović, Sanja Petrović, Aleksandra Popovac, Miloš Todorović, Aleksandra Milić-Lemić

**Affiliations:** 1 Department of Prosthodontics, School of Dental Medicine, University of Belgrade, Rankeova 4, 11000 Belgrade, Serbia; stefan.vulovic@stomf.bg.ac.rs (S.V.); aleksandra.popovac@stomf.bg.ac.rs (A.P.); 2 Department of Periodontology and Oral Medicine, School of Dental Medicine, University of Belgrade, Dr Subotica 4, 11000 Belgrade, Serbia; natasa.nikolic.jakoba@stomf.bg.ac.rs; 3 Department of Microbiology and Immunology, School of Dental Medicine, University of Belgrade, Dr Subotica 1, 11000 Belgrade, Serbia; milena.radunovic@stomf.bg.ac.rs (M.R.); sanja.petrovic@stomf.bg.ac.rs (S.P.); 4 Department of Pediatric and Preventive Dentistry, School of Dental Medicine, University of Belgrade, Dr Subotica 11, 11000 Belgrade, Serbia; milos.todorovic@stomf.bg.ac.rs

**Keywords:** polymers, resin-based composite, polymethyl methacrylate, polyether ether ketone, surface roughness, contact angle, colony forming unit, scanning electron microscopy

## Abstract

Dental polymers are now available as monolithic materials which can be readily used in computer-aided design and computer-aided manufacturing (CAD/CAM) systems. Despite possessing numerous advantages over conventionally produced polymers, the polymers produced by either of these systems fail to exhibit immunity to surface microbial adhesion when introduced into the oral environment, leading to the development of oral diseases. The aim of this study was to analyze the biofilm formation of six microorganisms from the oral cavity and its correlation to the surface characteristics of CAD/CAM dental polymers. A total of ninety specimens were divided into three groups: resin-based composite, polymethyl methacrylate, and polyether ether ketone. The experimental procedure included surface roughness and water contact angle measurements, colony forming unit counting, and scanning electron microscopy analysis of biofilm formed on the surface of the tested materials. The data were analyzed using the Kruskal–Wallis test, with a Dunn’s post hoc analysis, and one way analysis of variance, with a Tukey’s post hoc test; the correlation between the measurements was tested using Spearman’s correlation coefficient, and descriptive statistics were used to present the data. Despite using the same manufacturing procedure, as well as the identical manufacturer’s finishing and polishing protocols, CAD/CAM dental polymers revealed significant differences in surface roughness and water contact angle, and the increased values of both parameters led to an increase in biofilm formation on the surface of the materials. The CAD/CAM resin-based composite showed the lowest number of adhered microorganisms compared to CAD/CAM polymethyl methacrylate and CAD/CAM polyether ether ketone.

## 1. Introduction

Dental polymeric materials of different matrix compositions, with added resin or ceramic fillers, are now available as monolithic materials, ready for use in computer-aided design and computer-aided manufacturing (CAD/CAM) systems [1]. The most frequently used CAD/CAM polymers are resin-based composites (RBC), indicated for provisional fixed restorations; polymethyl methacrylate (PMMA), preferred for removable dentures and provisional fixed restorations; and polyether ether ketone (PEEK), used in a wide range of dental applications, including the manufacturing of removable dentures, short-span fixed dentures, abutments, and substructures in implant-supported restorations.

With the innovation of CAD/CAM technology, materials are pre-prepared in a standardized block or disc form, omitting the dependence on manual skills during the fabrication process. Thus, significantly enhanced mechanical and esthetic properties of the material are achieved, preserving the patient’s sense of taste and providing easier patient adaptation to these types of restorations [2,3]. Furthermore, the avoidance of the manual mixing of powder and liquid decreases the amount of released monomer and reduces the porosity of the CAD/CAM polymers [4]. Optimizing the mechanical properties leads to lower microbial adhesion and the diminished potential for allergic reactions, which contributes to material biocompatibility and the targeted indication for long-term dental restorations. However, none of these polymers, when introduced into the oral environment, is immune to surface microbial adhesion, the process that triggers material biodegradation. This process compromises the integrity of the dental restoration by facilitating liquid absorption, staining [5], the loss of gloss, and the development of oral diseases, such as secondary caries, periodontal diseases, and implant- or denture-related infections [6]. The initial microbial colonization on dental polymers begins at surface irregularities, where microorganisms are protected against shear forces, which allows them to create irreversible attachment to the material’s surface, providing conditions for secondary colonization by other species [7]. The material’s wettability might be another contributing factor responsible for microbial adhesion, suggesting that materials with higher surface hydrophilia are prone to microbial colonization [8]. Furthermore, the composition of the polymeric material itself may have an impact on bacterial adhesion, since dental polymers are comprised of many different ingredients, such as an organic matrix, added resin or ceramic particles, fluoride, and metal ions, that when combined, create a heterogeneous surface that is difficult to polish, leading to higher susceptibility for microbial adhesion [9].

Unlike conventionally produced dental polymers, which have been significantly researched up until now, to the knowledge of the authors, the present study is the first one focused on the characteristics of biofilm formed on the surfaces of CAD/CAM polymers and its relationship with the surface characteristics of the materials. Considering the role of the main etiological agent in the occurrence of caries [10], the biofilm of *Streptococcus mutans* was analyzed. Since the initial adhesion and the understanding of microorganism–surface interaction is essential for biofilm control and survival rate of the restoration [11], the present research was also focused on the adhesion of *Streptococcus oralis*, one of the primary colonizers that adheres directly to the soft and hard tissues in the oral cavity, creating conditions for the binding of other pathogens [12]. Furthermore, the study included analysis of *Veillonella parvula,* an intermediate colonizer capable of easily co-aggregating with streptococci [13], which is strongly associated to periodontal and peri-implant infections [14]. *Fusobacterium nucleatum* was introduced due to its important role in the pathogenesis of periodontitis, as it directly connects primary colonizers, such as representatives of the genus *Streptococcus*, with anaerobic secondary colonizers, such as *Porphyromonas gingivalis* [15]. As a secondary colonizer capable of adhering to a variety of host cells, producing several virulence factors [16] and acting as the major etiologic agent in severe chronic periodontitis and peri-implantitis, *Porphyromonas gingivalis* could not be overlooked. Moreover, for comprehensive study, the present research evaluated the adhesion of *Candida albicans*, the most established and described opportunistic microorganism in the oral cavity, highly related to periodontal, peri-implant, and denture-related infections.

The present research was performed with the aim of analyzing the biofilm formation of six different microorganisms from the oral cavity and its correlation to the surface characteristics of CAD/CAM dental polymers. The following null hypotheses were: 1. No correlation would be found between the materials’ surface roughness and the tested species’ biofilm formation. 2. No correlation would be found between the materials’ surface wettability and the tested species’ biofilm formation. 3. No significant difference would be found regarding the tested species’ biofilm formation among the investigated materials.

## 2. Materials and Methods

### 2.1. Specimen Preparation

**Figure 1:**
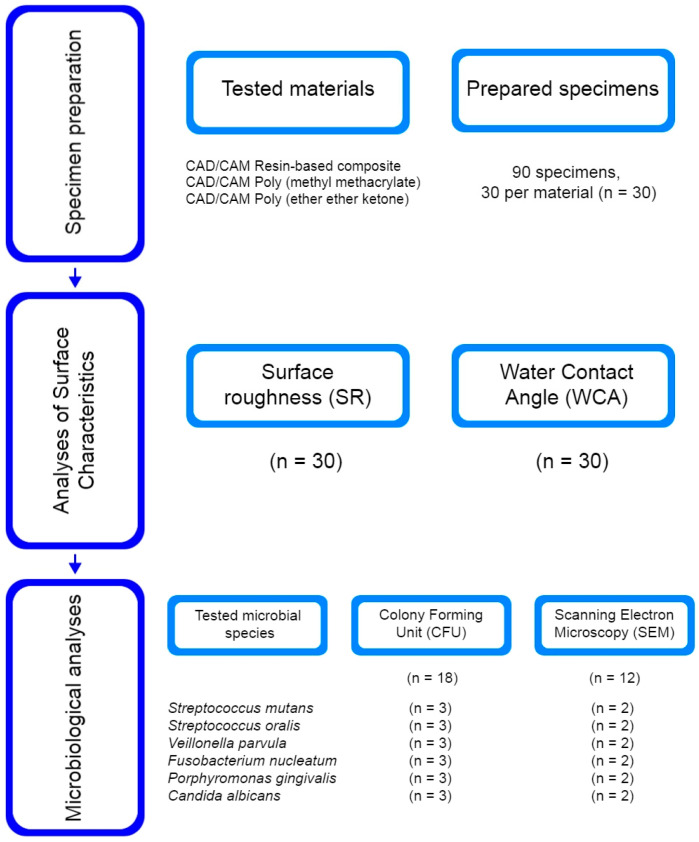
Flow chart of the study protocol with number of used specimens for each analysis.

The flow chart of the study protocol is illustrated in Figure 1. The experimental procedure included different CAD/CAM dental polymers (Table 1) divided into three groups: a resin-based composite (RBC), polymethyl methacrylate (PMMA), and polyether ether ketone (PEEK). A total of 90 specimens, 30 specimens per group (*n* = 30), was determined based on sample size calculations using the G*Power 3.1.9.7 program (Heinrich Heine University, Düsseldorf, Germany). According to the International Organization for Standardization (ISO) 20795-1:2013 [17], the materials were fabricated into disc-shaped specimens (5 mm diameter and 2 mm thickness), finished, and polished by the same operator, following the manufacturer’s instructions (Table 2), as described in a previous study [18]. After cleaning in an ultrasonic bath (Baku BK-3A, Baku, Guangzhou, China), in 70% ethanol for 5 min and distilled water for 5 min, the specimens were gently dried and exposed to ultraviolet light at room temperature for 30 min per side. The prepared specimens were stored in sterile microcentrifuge tubes until analysis.

### 2.2. Surface Roughness (SR)

Surface roughness (SR) measurements were performed using a profilometer (TR200, Beijing TIME High Technology, Beijing, China) on all specimens from each group (*n* = 30) using the 5 μm diamond stylus, with 1.25 mm total length, 0.25 mm cut-off value, 0.02 μm resolution, and a Gaussian filter, according to ISO 4288:1996 [19]. The arithmetic mean value of all peaks and valleys (Ra) in the measured profile was analyzed, and the average value from three measurements in three different directions was calculated for each specimen.

### 2.3. Water Contact Angle (WCA)

In order to evaluate the material’s surface wettability, the contact angle measurements of distilled water were conducted on all specimens from each group (*n* = 30). The measurements were performed under room temperature, 23 °C ± 2 °C, and humidity, 40% ± 2%, using the sessile drop technique [20]. After dispensing a drop (2 μL) of distilled water from a micropipette (BIOHIT, BiohitOyj, Helsinki, Finland), the contact between the drop and the material’s surface was photographed using a camera (Nikon D5200, Nikon, Minato, Tokyo, Japan) with a mounted lens (AF-S DX Nikkor Micro 85 mm f/3.5G ED VR, Nikon, Minato, Tokyo, Japan) and a flashlight (Sigma EM-140 DG, Sigma, Kawasaki, Kanagawa, Japan). The camera was fixed on a support, with specimens positioned at the standardized location, thus enabling equal conditions for each image taken. The obtained photos were then analyzed using ImageJ software (Version 1.42, National Institute of Health, Bethesda, MD, USA), where the angle of the tangent of a water drop to the material’s surface was measured on both left and right sides, yielding the average value for each specimen.

### 2.4. Biofilm Formation

Before microbiological analyses, the specimens underwent the same cleaning procedure as that employed before the surface roughness and water contact angle measurements. The research included reference strains of six microorganisms: *Strep. mutans* ATCC 25175, *Strep. oralis* ATCC 6249, *V. parvula* ATCC 10790, *F. nucleatum* ATCC 25586, *P. gingivalis* ATCC 332787, and *C. albicans* ATCC 10231 (Microbiologics KWIK-STIK, Manassas, VA, USA). Following the strain activation process (Table 3), 3–4 colonies of each species were transmitted to specific media: *Strep. mutans*, *Strep. oralis*, and *V. parvula* to Brain Heart Infusion (BHI) broth (HiMedia, Mumbai, India); *F. nucleatum* and *P. gingivalis* to Schaedler broth with hemin and vitamin K_1_ (Becton, Dickinson and Company, Franklin Lakes, NJ, USA); and *C. albicans* to Sabouraud broth (HiMedia, Mumbai, India), all incubated under the previously presented growth conditions (Table 3). After subjecting the obtained bacterial/fungal suspension to a centrifugation process (10 min, 3000 rpm), sterile phosphate-buffered saline (PBS) was added to each suspension (turbidity of 1.0 McFarland standard, ≈10^8^ cells/mL for bacteria, and ≈10^6^ cells/mL for *C. albicans*) (DEN-1 densitometer, Biosan, Riga, Latvia). The suspensions were then diluted with BHI broth for *Strep. mutans*, *Strep. oralis*, and *V. parvula;* with Schaedler broth with hemin and vitamin K1 for *F. nucleatum* and *P. gingivalis;* and with RPMI 1640 medium with 2% glucose (Sigma-Aldrich, St. Louis, MO, USA) for *C. albicans*, adjusting the microorganism number to 10^6^ for bacteria and 10^5^ for *C. albicans*. Biofilms were created on all specimens from each group (*n* = 30), five per each of the six species. After inserting the specimens into 96-well plates, 150 μL of saliva was added to each well in order to form a primary pellicle. Following the 24 h incubation process at 37 °C, the saliva was eliminated, and 200 μL of bacterial/fungal suspension was added to each well. The wells were then incubated under the following conditions: *Strep. mutans* and *Strep. oralis* were incubated for 24 h at 37 °C under anaerobic conditions; *V. parvula*, *F. nucleatum*, and *P. gingivalis* were incubated for 5 days at 37 °C under anaerobic conditions; and *C. albicans* was incubated for 48 h at 37 °C under aerobic conditions.

### 2.5. Colony Forming Unit (CFU)

The number of colonies per mL (CFU/mL) was counted for eighteen specimens from each group (*n* = 18), three per each species. Aiming to eliminate unattached bacterial/fungal cells, the specimens were washed in sterile PBS and then transferred into sterile microcentrifuge tubes containing 1 mL of sterile PBS; thereafter, each tube was cleaned in an ultrasonic bath for 1 min at 40 kHz and treated in a shaking device (Varioshake VS 15B, Lauda, Lauda-Königshofen, Germany) for 15 min at 37 °C and 900 rpm. Serial ten-fold dilutions of PBS from the tubes were seeded on previously presented media (Table 3). The plates were then incubated at 37 °C, under anaerobic (for bacterial species) or aerobic conditions (for *C. albicans*), for 24 h (for *Strep.* mutans, *Strep. oralis*, and *C. albicans*), or 5 days (for *V. parvula*, *F. nucleatum* and *P. gingivalis*). After incubation, the amount of biofilm was determined and expressed as CFU/mL.

### 2.6. Scanning Electron Microscopy (SEM)

A scanning electron microscope (JEOL JSM-6610LV, Jeol, Akishima, Tokyo, Japan) was used for the two-dimensional (2D) display of biofilm formed on the surfaces of the specimens. The evaluation process was performed on twelve specimens per each group (*n* = 12), two per each species. For the proper SEM procedure, the biofilm-covered specimens were subjected to the following preparation protocol [21]:

- Rinsing the specimens with sterile PBS in order to remove detached cells.
- Immersion of the specimens in 2.5% glutaraldehyde for 48 h in order to fix the biofilm on the surface of the specimen.
- Dehydration of the specimens using increased ethanol concentrations (50%, 60%, 70%, 80%, 90%, and 100%) in 3% acetic acid solution for 1 h.
- Drying the specimens in a critical point dryer using carbon dioxide (CO_2_).
- Coating the specimens with a 20 nm layer of gold for 2 min to guarantee the conductivity of electrons, prevent electrical charge build-up within a specimen, and improve micrograph resolution.
- Scanning the specimens with a device operating at 20 kV, with tilt angles ranging from 10° to 45°, and at ×500 and ×3500 magnification.

### 2.7. Statistical Analysis

The data analysis was performed using statistical software (SPSS v22.0, SPSS, Chicago, IL, USA) with α = 0.05 level of statistical significance and 80% statistical power. After testing the normality of data using the Kolmogorov–Smirnov test, Ra and WCA data revealed a non-normal distribution and were submitted to a comparison among groups using the Kruskal–Wallis test, with a Dunn’s post hoc analysis. CFU/mL data for all species were normally distributed and thus, compared among groups using one way analysis of variance (ANOVA), followed by Tukey’s post hoc test. Spearman’s rank correlation coefficient was used for the interrelation between Ra and CFU/mL and between WCA and CFU/mL for all species. Ra and WCA data were presented with median (min–max), and CFU/mL data for all species were presented as mean ± standard deviation (SD).

## 3. Results

### 3.1. Surface Roughness (SR)

The results of SR measurements (Table 4) revealed a significantly lower Ra value in the PMMA group, compared to both the RBC (*p* = 0.001) and PEEK specimen groups (*p* < 0.001). Between RBC and PEEK, no statistically significant difference was observed (*p* = 0.211), with a slightly higher Ra value in the PEEK group.

### 3.2. Water Contact Angle (WCA)

**Figure 2:**
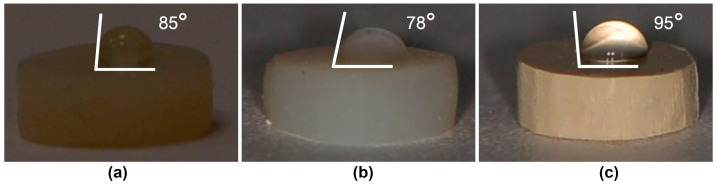
Water contact angle analysis on representative specimens from each group: (**a**) resin-based composite (RBC); (**b**) polymethyl methacrylate (PMMA); (**c**) polyether ether ketone (PEEK).

The obtained images from the WCA measurements of representative specimens from each group are presented in Figure 2, with values summarized in Table 4. Statistical analysis revealed a significantly higher value in the PEEK group, compared to both the RBC (*p* < 0.001) and PMMA groups (*p* < 0.001). Comparing PMMA and RBC, an insignificantly lower value (*p* = 0.054) was observed in the PMMA group.

### 3.3. Colony Forming Unit (CFU)

**Figure 3:**
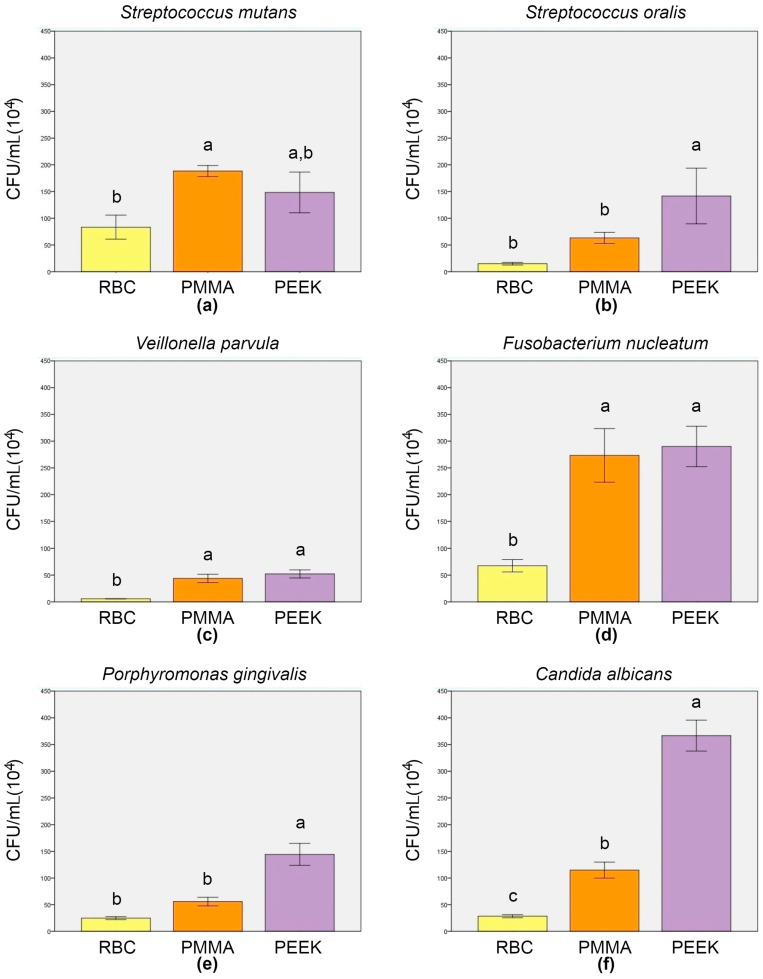
Colony forming unit analysis results (mean ± SD) of adhered *Strep. mutans* (**a**), *Strep. oralis* (**b**), *V. parvula* (**c**), *F. nucleatum* (**d**), *P. gingivalis* (**e**), and *C. albicans* (**f**). Error bars represent the ± SD, and different letters above SD indicate a significant difference among the groups (*p* < 0.05; Tukey’s post hoc test). RBC = resin-based composite; PMMA = polymethyl methacrylate; PEEK = polyether ether ketone.

The CFU counting results are presented in Figure 3. The *Strep. mutans* CFU/mL results (Figure 3a) revealed a significantly higher mean value for the biofilm grown on PMMA specimens (188.33 ± 10.41 × 10^4^), compared to RBC (83.33 ± 22.55 × 10^4^; *p* = 0.007), and an insignificantly higher mean value, compared to PEEK (148.33 ± 38.19 × 10^4^; *p* = 0.229). Between RBC and PEEK, no significant difference was observed (*p* = 0.053). The amount of adhered *Strep. oralis* colonies (Figure 3b) on PEEK (141.67 ± 52.04 × 10^4^) was significantly higher compared to RBC (15 ± 2.5 × 10^4^; *p* = 0.006) and PMMA (63.33 ± 10.41 × 10^4^; *p* = 0.046). Between RBC and PMMA, the absence of a significant difference was noticed (*p* = 0.211). It is clearly seen (Figure 3c) that RBC dominated, with a significantly lower *V. parvula* mean value (5.83 ± 0.29 × 10^4^), compared to both PMMA (43.83 ± 7.85 × 10^4^; *p* = 0.001) and PEEK (52.33 ± 7.50 × 10^4^; *p* < 0.001), between which no significant difference was detected (*p* = 0.294). *F. nucleatum* CFU/mL results (Figure 3d) revealed similar mean values for PEEK (290 ± 37.75 × 10^4^) and PMMA (273.33 ± 50.08 × 10^4^; *p* = 0.848). Furthermore, both groups revealed significantly higher values, compared to RBC (67.5 ± 11.46 × 10^4^; *p* = 0.001). Significant differences in CFU/mL values were found among the groups for both *P. gingivalis* (Figure 3e) and *C. albicans* (Figure 3f). The highest amount of both species was observed on the surfaces of the PEEK specimens (144.5 ± 20.5 × 10^4^ for *P. gingivalis* and 366.67 ± 28.87 × 10^4^ for *C. albicans*), compared to RBC (24.83 ± 2.75 × 10^4^; *p* < 0.001 for *P. gingivalis* and 28.33 ± 2.89 × 10^4^; *p* < 0.001 for *C. albicans*) and PMMA (55.83 ± 8.04 × 10^4^; *p* < 0.001 for *P. gingivalis* and 115 ± 15 × 10^4^; *p* < 0.001 for *C. albicans*). Compared to RBC, PMMA values were insignificantly higher for *P. gingivalis* (*p* = 0.057) and significantly higher for *C. albicans* (*p* = 0.003). Spearman’s rank correlation coefficient revealed a positive interrelation between Ra and CFU/mL and between WCA and CFU/mL for all tested species (Table 5).

### 3.4. Scanning Electron Microscopy (SEM)

**Figure 4:**
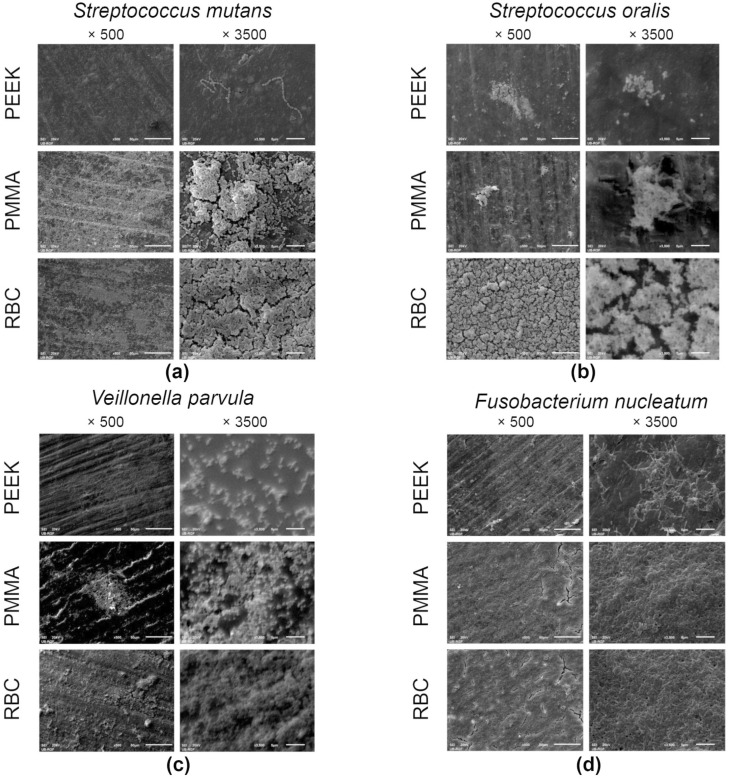

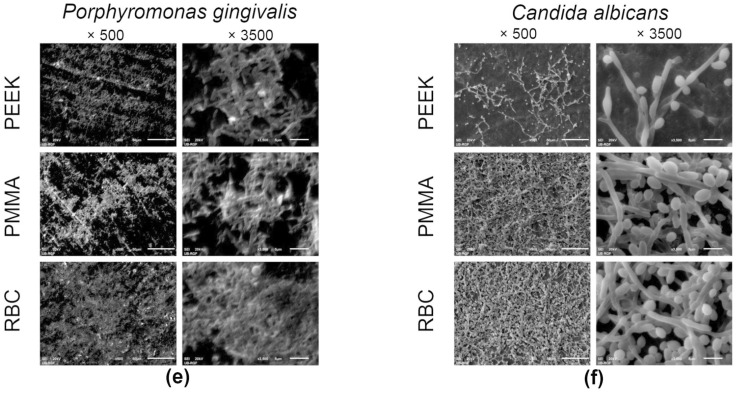
Scanning electron microscopy micrographs of adhered *Strep. mutans* (**a**), *Strep. oralis* (**b**), *V. parvula* (**c**), *F. nucleatum* (**d**), *P. gingivalis* (**e**), and *C. albicans* (**f**) on representative specimens from each group at ×500 and ×3500 magnifications. RBC = resin-based composite; PMMA = polymethyl methacrylate; PEEK = polyether ether ketone.

Representative SEM micrographs of the specimens covered with microorganisms are displayed in Figure 4. Recorded images of adhered *Strep. mutans* (Figure 4a) corroborate the CFU results by confirming the evidently denser layer of well-organized aggregates of cocci on the PMMA and PEEK specimens, compared to RBC, where only a few chain-grouped colonies covered the central part of the specimen. Micrographs of the adhered *Strep. oralis* (Figure 4b) revealed numerous cocci across the surface of the PEEK specimen. On the contrary, micrographs of RBC and PMMA specimens showed a few cluster-shaped colonies, surrounded by a relatively homogenous surface texture, with a couple of longitudinal grinding grooves. A biofilm of *V. parvula* (Figure 4c) was not clearly observed on the surfaces of specimens at lower magnifications. However, a closer view enabled the clear characterization of small, round-shaped microorganisms across the surfaces of the PEEK and PMMA specimens. The RBC specimen revealed a minor portion of cocci, surrounded by areas without microorganisms in the background. Figure 4d presents the domination of spindle-shaped *F. nucleatum* over the entire surfaces of the PMMA and PEEK specimens, at both magnifications. On the other hand, RBC micrographs enabled a clear view of the *F. nucleatum* grouping pattern by demonstrating a few elongated bacilli, mostly connected in pairs. Similar observations were found for the images with the *P. gingivalis* biofilm (Figure 4e), where rod-shaped bacteria are spread across the PEEK surface. The PMMA and RBC micrographs exhibited a high degree of similarity, enabling a view of the linearly-distributed microorganisms. The *C. albicans* micrographs (Figure 4f) revealed that the biofilm mass almost completely covered the surfaces of the PEEK and PMMA specimens, contrary to the micrographs of the RBC specimen, which showed a minor portion of chain-grouped fungi. Higher magnification enabled a clear characterization of pseudohyphal growth of *C. albicans* on all evaluated specimens.

## 4. Discussion

CAD/CAM dental polymers might be considered as potential materials for application in the creation of dental restorations with similar surface characteristics due to the standardized manufacturing process and similar polymeric composition patterns. However, the SR results from the present research revealed significantly lower values in the PMMA group, compared to the RBC and PMMA groups. This result is in agreement with the results from previous studies, in which PMMA was considered as relatively smooth [22], in contrast to RBC and PEEK, which were described as materials filled with different particles, presenting a heterogeneous surface morphology which is difficult to polish [18,23,24]. All tested materials revealed Ra values above 0.2 μm, which was considered as the threshold value below which the role of surface irregularities in regards to plaque adherence on material’s surface is eliminated [25]. In accordance with the statement, the present study found a very weak positive correlation between Ra and the CFU/mL of *Strep. mutans*, *F. nucleatum*, and *C. albicans*, and a weak positive correlation between Ra and the CFU/mL of *Strep. oralis*, *V. parvula*, and *P. gingivalis*, suggesting the increase in microbial adhesion on rougher surfaces. Therefore, the first null hypothesis is rejected. There is a lack of research investigating the influence of surface characteristics on microbial adhesion on CAD/CAM dental polymers. Numerous studies focused on conventionally produced materials suggested that the rougher surfaces of RBC [24], PMMA [26], and PEEK [27] promote the increase in biofilm formation. Investigating the same microorganisms, several authors found a strong linear correlation between the SR of RBC and the adhesion of oral streptococci [28] and between the SR of PMMA and *C. albicans* biofilm [29,30]. On the other hand, the results from the present study are in disagreement with those of other authors, who did not find a clear relationship between the SR of RBC and the number of adhered *Strep. mutans* [31], *Strep. oralis* [32], and *C. albicans* [33], or between the SR of PMMA and *Strep. mutans* [34,35] or *C. albicans* adhesion [36]. Furthermore, there are only a few studies investigating the adhesion of *V. parvula*, *F. nucleatum*, or *P. gingivalis* on dental polymers, and the results of these studies indicated that the adhesion of *P. gingivalis* was not significantly influenced by SR [37].

Water contact angle measurements enabled the characterization of the material’s surface wettability. The present study revealed WCA values lower than 90° in both the RBC and PMMA specimens, correlating these groups with hydrophilic behavior and a WCA value more than 90° only in the PEEK group. These results describe this material as hydrophobic [38], which is in agreement with the results of previous investigations that attributed PEEK’s hydrophobic behavior to the nonpolar functional groups contained in the structure of PEEK [25,39,40]. The reported very weak positive correlation between the WCA and CFU/mL of *Strep. mutans*, the weak positive correlation between the WCA and CFU/mL of *F. nucleatum* and *P. gingivalis*, and the moderate positive correlation between the WCA and CFU/mL of *Strep. oralis*, *V. parvula*, and *C. albicans* suggest that the tested materials exhibiting higher contact angle values and greater hydrophobic behavior are likely to exhibit increased microbial colony adhesion. Therefore, the second null hypothesis is also rejected. Previous studies on conventionally produced polymers [31,41,42,43,44,45,46,47,48] have presented conflicting results and, until now, no clear relationship between a material’s surface wettability and biofilm formation on the surfaces of dental polymers has been revealed. The results from the present study are in agreement with those of several authors, who described *Strep. oralis* [41] and *C. albicans* [42] as hydrophobic microbial strains, preferentially adhering to more hydrophobic surfaces. Another assumption is that the higher surface hydrophilia of polymeric materials leads to increased bacterial colonization on their surfaces. This has been shown in previous studies, based on the adherence of oral streptococci on the surfaces of RBC [43] and PEEK [44], *F. nucleatum* on the surface of PMMA [45], and *C. albicans* on the surfaces of RBC [46] and PMMA [47]. However, numerous researchers found no correlation between the material’s surface wettability and microbial adhesion on RBC [31] or PMMA [48], rejecting the direct influence of the described parameter on microbial adhesion on the material’s surface.

The lack of a strong correlation between the reported surface roughness and the water contact angle with the analyzed microbial adhesion in the present study confirmed the premise that surface characteristics are not always crucial, from a microbiological standpoint, and that the biofilm formation on CAD/CAM dental polymers is additionally affected by the material’s chemical composition. Furthermore, CFU counting, supported by SEM analysis, revealed significant differences in the amount of all tested microbial species among the material groups. Therefore, the third null hypothesis is also rejected. Although numerous studies marked RBC as a material susceptible to microbial colonization, due to polymerization shrinkage, the leakage of unpolymerized monomers, and the biodegradation products emitting from the material’s surface [49,50], the results from the present research support the fact that the introduction of nanotechnology and CAD/CAM systems significantly improved the RBC characteristics from a microbiological aspect, and that composite resins are more resistant to attack by oral microbiota [45,51]. Finally, PEEK was introduced as the material most susceptible to microorganisms. This result was also shown in previous similar studies comparing PEEK’s antimicrobial behavior with the behavior of other materials, using SEM [27] or crystal violet stain analysis for biofilm characterization [52]. However, the results from the present study are in disagreement with those of other authors, who labelled PEEK as possessing excellent antibacterial properties against oral microorganisms after analyzing the relative number of viable microorganisms present using a cell viability assay [53]. These contradictory outcomes might be explained by the different methodological designs implemented in each study.

Although the design of the present research has been implemented in numerous previous studies [34,41,54,55,56], the major limitation of the present in vitro research was the impossibility of completely reproducing the conditions in the oral cavity, where the adhesion and development of certain species on the surfaces of dental materials is highly affected by the presence of other microorganisms. Furthermore, during the aging process in the oral cavity, after been subjected to temperature changes and chewing, the surface roughness of the materials inevitable alters, consequently affecting microbial adhesion [57]. Moreover, this study did not involve the use of a bioreactor with a continuous flow of the nutrient medium through the flow cells, which presents another drawback. Finally, the reported surface characteristics, which were correlated with microbial adhesion, were obtained using only a profilometer surface roughness analysis and contact angle measurements, without a detailed characterization of the surface using SEM 2D surface texture and fractal analysis, or atomic force microscopy (AFM) 3D analysis [58], providing only a partial relationship between the surface quality and microbial adhesion. A good understanding of the complexity of oral biofilm is of crucial importance for the adequate prevention of oral diseases and the improvement of overall health. Therefore, it is vital to create a CAD/CAM dental polymeric material, which, besides providing numerous advantages over conventionally produced materials, reveals excellent antimicrobial properties. Although the results from the present study may contribute to this goal by enabling a closer view into some characteristics of the tested materials, creating such a material that fulfills all the necessary requirements remains a serious challenge. Therefore, additional in situ studies are necessary to confirm the susceptibility of the tested materials to microbial adhesion under the dynamic conditions of the oral environment.

## 5. Conclusions

Based on the findings of the current study, it was concluded that:

1. The increase in surface roughness leads to an increase in biofilm formation on the surfaces of CAD/CAM dental polymers.
2. The increase in water contact angle and the material’s hydrophobicity leads to an increase in biofilm formation on the surfaces of CAD/CAM dental polymers.
3. The CAD/CAM resin-based composite is less susceptible to microbial adhesion compared to the CAD/CAM polymethyl methacrylate and CAD/CAM polyether ether ketone.

## Figures and Tables

**Table 1:** Materials used in the study.

Material	Group	Chemical Composition	Brand Name	Manufacturer
Resin-based composite	RBC	27 wt.% inorganic fillers in a polymer matrix	Structur CAD	VOCO, Cuxhaven, Germany
Polymethyl methacrylate	PMMA	Double cross-linked polymethyl methacrylate	Ivotion dent	Ivoclar Vivadent, Schaan, Liechtenstein
Polyether ether ketone	PEEK	20 wt.% ceramic filler, grain size 0.3 μm–0.5 μm	breCAM.BioHPP	Bredent group, Senden, Germany

**Table 2:** Specimen preparation procedure.

Material	Material Dimensions	Cutting Procedure	Finishing and Polishing Procedure	Specimens Dimensions
Resin-based composite	Disc-shaped (diameter 98.4 mm, thickness 20 mm)	Diamond blade (15LC, Buehler, Lake Bluff, IL, USA) in a cutting machine (Isomet 4000, Linear Precision Saw, Buehler, Lake Bluff, IL, USA)	1. Rubber polisher (Politip Polisher Refill x6, Ivoclar Vivadent, Schaan, Liechtenstein) 2. Goat hair brush (Polishing Brush 110 104 190 White/5, Meisinger, Ivoclar Vivadent, Schaan, Liechtenstein), in combination with polishing paste (Universal Polishing Paste, Ivoclar Vivadent, Schaan, Liechtenstein)	Disc-shaped (diameter 5 mm, thickness 2 mm)
Polymethyl methacrylate	Disc-shaped (diameter 98.5 mm, thickness 20 mm)	Diamond blade in a cutting machine	1. Leather brush (Polishing Brush 140 104 220 Gray Leather/5, Meisinger, Ivoclar Vivadent, Schaan, Liechtenstein) 2. Abrasive rubber point (Abraso-Gum Acrylic Polisher medium REF P243HM10, Bredent group, Senden, Germany) 3. Lathe brush (Polishing Brush B27 Wood Center 207-0027, Rite Dent, Sialkot, Pakistan), in combination with pumice powder (PoloDent Pumice Powder, Polo MB, Oisterwijk, The Netherlands) 4. Cotton buff (Polishing Brush 150 104 220 Cotton White/5, Meisinger, Ivoclar Vivadent, Schaan, Liechtenstein), in combination with polishing paste	Disc-shaped (diameter 5 mm, thickness 2 mm)
Polyether ether ketone	Disc-shaped (diameter 98.5 mm, thickness 20 mm)	Diamond blade in a cutting machine	1. Tungsten-carbide bur (HM cutter 2.3 mm Ø with conical round, REF H200M823, Bredent group, Senden, Germany) 2. Two types of rubber points (Abraso-Gum Acrylic Polisher, rough REF P243HG10, and medium REF P243HM10, Bredent group, Senden, Germany)	Disc-shaped (diameter 5 mm, thickness 2 mm)

**Table 3:** Growth conditions for activation of reference microbial strains.

Reference Strain	Growth Medium	Temperature	Time	Conditions
*Streptococcus mutans* ATCC 25175	Mutans-Sanguis agar (HiMedia, Mumbai, India)	37 °C	24 h	Anaerobic
*Streptococcus oralis* ATCC 6249	Columbia agar with 5% sheep blood (ProReady, Kikinda, Serbia)	37 °C	24 h	Anaerobic
*Veillonella parvula* ATCC 10790	Brain Heart Infusion (BHI) agar with 5% sheep blood (HiMedia, Mumbai, India)	37 °C	24 h	Anaerobic
*Fusobacterium nucleatum* ATCC 25586	Brucella agar with 5% sheep blood, hemin, and vitamin K_1_ (Becton, Dickinson and Company, Franklin Lakes, NJ, USA)	37 °C	24 h	Anaerobic
*Porphyromonas gingivalis* ATCC 332787	Brucella agar with 5% sheep blood, hemin, and vitamin K_1_ (Becton, Dickinson and Company, Franklin Lakes, NJ, USA)	37 °C	24 h	Anaerobic
*Candida albicans* ATCC 10231	Sabouraud Agar (HiMedia, Mumbai, India)	37 °C	24 h	Aerobic

**Table 4:** Results of the surface roughness (Ra) and water contact angle (WCA) analyses.

Specimen Group	Ra (μm) (Median; Min–Max)	WCA (°) (Median; Min–Max)
RBC	0.32; 0.24–0.37 ^a^	82.07; 71.51–89.99 ^b^
PMMA	0.28; 0.21–0.33 ^b^	79.49; 69.65–84.43 ^b^
PEEK	0.33; 0.25–0.40 ^a^	96.02; 86.79–99.44 ^a^

Note: Different superscript letters indicate statistically significant difference inside the respective column; *p* < 0.05 (Dunn’s post hoc test).

**Table 5:** Spearman’s rank correlation coefficient results.

Correlated Data	ρ Value	Correlation
Ra—CFU/mL *Strep. mutans*	0.143	very weak positive
Ra—CFU/mL *Strep. oralis*	0.288	weak positive
Ra—CFU/mL *V. parvula*	0.392	weak positive
Ra—CFU/mL *F. nucleatum*	0.169	very weak positive
Ra—CFU/mL *P. gingivalis*	0.343	weak positive
Ra—CFU/mL *C. albicans*	0.178	very weak positive
WCA—CFU/mL *Strep. mutans*	0.150	very weak positive
WCA—CFU/mL *Strep. oralis*	0.561	moderate positive
WCA—CFU/mL *V. parvula*	0.418	moderate positive
WCA—CFU/mL *F. nucleatum*	0.393	weak positive
WCA—CFU/mL *P. gingivalis*	0.367	weak positive
WCA—CFU/mL *C. albicans*	0.529	moderate positive

## Data Availability

The data presented in this study are available on request from the corresponding author.
